# Temperatures of storage areas in large animal veterinary practice vehicles in the summer and comparison with drug manufacturers’ storage recommendations

**DOI:** 10.1186/s12917-015-0561-z

**Published:** 2015-10-01

**Authors:** Jeff D. Ondrak, Meredyth L. Jones, Virginia R. Fajt

**Affiliations:** Great Plains Veterinary Educational Center, University of Nebraska-Lincoln, Clay Center, NE USA; Large Animal Clinical Sciences, Texas A&M University College of Veterinary Medicine and Biomedical Sciences, College Station, TX USA; Veterinary Physiology and Pharmacology, Texas A&M University College of Veterinary Medicine and Biomedical Sciences, College Station, TX USA

**Keywords:** Pharmaceuticals, Drug storage, Large animal practice, Drug stability, Excessive heat

## Abstract

**Background:**

Large animal veterinarians carry drugs in their practice vehicles in storage areas that are not typically refrigerated. The most common upper limits of manufacturers’ storage temperatures for United States (U.S.)-approved non-refrigerated drugs are 25 or 30 °C. Because ambient temperatures in many locations in the U.S. exceed these temperatures during the summer, we measured storage area temperatures over 4 months in the summer of 2013 to evaluate the extent to which labeled storage temperatures are exceeded.

**Methods:**

A convenience sample of 12 vehicles from 5 central Texas practices and 12 vehicles from 4 south central Nebraska practices was used. Temperatures were recorded in one drug storage compartment in each vehicle from May 15 – September 16, 2013, at 15-minute intervals using a self-contained, battery operated temperature recording device.

**Results:**

The highest temperatures recorded in a storage unit were 54.4 and 47.7 °C in Texas and Nebraska, respectively. The mean temperature recorded across all 24 storage units was 29.1 °C, with a mean of 26.9 °C in Nebraska and 31.4 °C in Texas. In Nebraska, at least one temperature over 25 °C was recorded on a mean of 111/124 days and a mean of 63 % of total logger readings. In Texas, temperatures over 25 °C were recorded on a mean of 123/124 days and a mean of 95 % of total logger readings.

**Conclusions:**

Temperatures in storage units in participating veterinary practice vehicles exceeded labeled drug storage temperatures a significant portion of the summer of 2013. More research is needed to determine whether these excursions above the manufacturers’ recommended storage temperatures alter efficacy of stored drugs.

## Background

Large animal veterinarians, those serving horses, cattle, and other livestock, often provide medical care by traveling to farms and other sites where animals are located. The travel vehicle is used to carry equipment, supplies, and drugs, and those items often remain in the truck at all times. These practice vehicles often have after-market storage areas installed, or storage compartments may be built into or added to the inside of the vehicle. These storage areas may be equipped with small refrigerators and heating capabilities, but generally refrigerator use is limited to items that have manufacturers’ requirements for storage at lower than room temperature (20–25 °C) [1].

The U.S. Pharmacopeia (USP) publishes guidelines for the pharmaceutical industry for potency, stability testing, and storage, including temperature. These guidelines apply to all aspects of the supply chain including transport vehicles, which include shipping vehicles and emergency medical service vehicles [2]. These guidelines state that ‘temperature is one of the most important conditions to control’ [2]. Other environmental factors that affect storage stability include light, air, and humidity [3].

Storage temperatures for drugs have been studied in human medical emergency service vehicles [4–7], medical helicopters [8], as well as medical bags [9], and storage container temperature frequently falls outside of label ranges. In a model of stock rotation based on actual measured temperatures in ambulances in five U.S. cities (Topeka, Orlando, Mesa, Portland and Syracuse), excessive heat occurred in all, including the northern cities [10]. Of concern, some emergency medications, including lorazepam [4, 7], have demonstrated instability at real-world ambulance temperatures. In experimental simulation of the ambulance environment, other emergency drugs including epinephrine, lidocaine, diltiazem, dopamine and nitroglycerin experienced a greater than 10 % reduction in concentration, which was correlated with thermal exposure time [11]. Based on the findings of these studies, the USP has added a section to the Good Storage and Distribution Practices for Drug Products which specifically addresses emergency medical service vehicles and other road vehicles used to transport drug products, indicating that temperature monitoring devices should be placed in different areas of the trunk or cabin for monitoring during seasonal extremes [2]. However, these practices do not include specific recommendations for veterinary practice vehicles.

We are acquainted with the storage options and storage practices of large animal veterinarians in the U.S. based on personal experience with practice vehicles (combined we have utilized practice vehicles ourselves from at least 7 different locations, including academic and private practice) and via professional networks. We became concerned that drugs that were stored in non-refrigerated areas were being subjected to temperatures significantly higher than room temperature. One small study of a veterinary vehicle in England evaluated the temperatures in the car and in a drug storage cabinet within the vehicle, where it was found that the cabinet heated more slowly than did the car, but also cooled more slowly [12], however, we could find no other published studies specific to veterinary practice vehicles.

Our review of drug labels of commonly used drugs in large animal veterinary practice demonstrated that the most common upper limits of storage temperature authorized on drug labels are 25 and 30 °C. Because ambient temperatures in the states in which we currently provide veterinary care consistently exceed these temperature during the summer, the objective of this study was to measure storage area temperatures from May to September, the hottest months of the year, in two distinct geographical areas (central Texas and south central Nebraska), to evaluate the extent to which manufacturers’ recommended storage temperatures were exceeded.

## Methods

### Selection of practice vehicles

A convenience sample of 12 vehicles from 5 central Texas veterinary practices and 12 vehicles from 4 south central Nebraska veterinary practices was used. Data on the veterinary practice type, storage unit characteristics, location of the temperature logger within the storage unit, and typical parking locations and conditions of the vehicles were collected with a handwritten survey.

### Storage unit temperature recording

Temperatures were recorded in one drug storage compartment in each vehicle for 124 days from May 15 – September 16, 2013, at 15-min intervals using a self-contained, battery-operated temperature recording device (HOBO Water Temperature Pro v2 Data Logger, Onset Computer Corporation, Cape Cod, MA). The temperature loggers were factory calibrated to +/− 0.2 °C accuracy.

### Ambient temperature source

Information regarding high and low ambient temperatures for the study period was obtained from Weather Underground^1^ for College Station and Navasota, Texas and Plymouth, Overton and Sutton, Nebraska. All veterinary units were based in locations within a 30 mile radius from one of these data centers.

### Descriptive statistics

Descriptive statistics were performed on the storage temperature data, survey responses and ambient temperature data using commercially available software (Excel, Microsoft Corporation, Redmond, WA and Graph Pad Prism, La Jolla, CA).

## Results

### Practice vehicle characteristics

Twenty four practice vehicles including 23 pick-up trucks with commercially available add-on storage units and one sport utility vehicle utilizing in-cabin storage were enrolled in the project. Participating practices self-identified by practice type as equine (*n* = 6), large animal (*n* = 1), food animal (*n* = 4), and mixed animal (*n* = 13) (Table 1). Of the 24 practice vehicles participating in this study, 18 of 24 (75 %) were subjected to routine unshaded conditions during working hours including 7 of 12 (58 %) in Texas and 11 of 12 (92 %) in Nebraska. Ten of 24 (42 %) vehicles were maintained in unshaded conditions during non-business hours including 7 of 12 (58 %) in Texas and 3 of 12 (25 %) in Nebraska.

**Table 1:** Characteristics of participating veterinary practice vehicles and storage units

Logger ID	Location		Practice type	Box information				Parking conditions	
	State	Clinic		Make	Refrigerator use	Day heater	Heated water	Home/Night	Clinic/Day
22	NE	1	Mixed	Porta-Vet	No	Off	Off	Unshaded	Unshaded
24	NE	1	Mixed	Porta-Vet	No	Off	Off	Unshaded	Unshaded
26	NE	2	Mixed	Bowie	Yes	Off	Off	Garage	Unshaded
36	NE	2	Mixed	Porta-Vet	No	On	On	Unshaded	Unshaded
43	NE	2	Mixed	Bowie	No	Off	Off	Garage	Unshaded
51	NE	2	Mixed	Bowie	Yes	On	On	Garage	Unshaded
58	NE	2	Mixed	Bowie	Yes	Off	On	Garage	Unshaded
46	NE	3	Food Animal	Porta-Vet	No	Off	On	Other shade	Unshaded
53	NE	3	Food Animal	Porta-Vet	No	Off	Off	Garage	Unshaded
57	NE	3	Mixed	Porta-Vet	No	Off	On	Garage	Unshaded
60	NE	3	Food Animal	Porta-Vet	No	Off	On	Garage	Unshaded
52	NE	4	Food Animal	Bowie	No	Off	On	Garage	Garage
33	TX	6	Mixed	Bowie	No	Off	Off	Carport	Carport
44	TX	6	Mixed	Porta = Vet	No	Off	Off	Unshaded	Unshaded
47	TX	6	Mixed	Bowie	No	Off	Off	Unshaded	Unshaded
49	TX	6	Mixed	Porta = Vet	No	Off	Off	Unshaded	Unshaded
56	TX	5	Equine	SUV cargo storage	No	No	No	Unshaded	Unshaded
62	TX	5	Equine	Bowie	No	Off	Off	Tree shade	Tree shade
42	TX	7	Equine	Bowie	Yes	Off	On	Carport	Carport
48	TX	7	Equine	Bowie	Yes	On	On	Carport	Carport
55	TX	7	Large Animal	Bowie	Yes	Off	On	Carport	Carport
40	TX	8	Equine	Stonewell Bodies	Yes	Off	Off	Unshaded	Unshaded
59	TX	8	Equine	Stonewell Bodies	Yes	Off	Off	Unshaded	Unshaded
39	TX	9	Mixed	Bowie	No	Off	Off	Unshaded	Unshaded

Small refrigerator units designed to store products specifically labelled for storage under refrigerated conditions and contained within the storage unit were in place and turned on in 8 of the 24 (33 %) practice vehicles including 5 of 12 (42 %) in Texas and 3 of 12 (25 %) in Nebraska. During the study period 4 of the 24 (17 %) storage unit’s internal heaters were reported to be set to the on position including 2 of 12 (17 %) in Texas and 2 of 12 (17 %) in Nebraska. Ten of the 24 participating veterinarians reported utilized the heated water feature of their storage units during the study including 3 of 12 (25 %) in Texas and 7 of 12 (58 %) in Nebraska.

### Storage unit temperature readings

The highest overall temperature recorded in a storage unit was 54.4 °C in a Texas vehicle and 47.7 °C in Nebraska (Table 2). The mean high temperatures were 41.3 and 43.9 °C for Nebraska and Texas, respectively, with the overall mean high temperature of 42.6 °C. The overall mean temperature in storage units was 26.9 °C in Nebraska and 31.4 °C in Texas. In Nebraska, at least one temperature over 25 °C was recorded on a mean of 111/124 days and a mean of 63 % of total logger readings. In Texas, temperatures over 25 °C were recorded on a mean of 123/124 days and a mean of 95 % of total logger readings. At least one reading over 30 °C was recorded a mean of 74/124 days in Nebraska and 117/124 days in Texas.

**Table 2:** Temperature logger and ambient temperature summary data

Source	Clinic	Low (°C)	High (°C)	Mean (°C)	% readings > 25 °C	% days > 25 °C	% readings > 30 °C	% days > 30 °C	Total number of readings
Logger 22	1	11.2	41.2	25.1	48.6	90	18.8	57	11,904
Logger 24	1	10.4	37.9	24.6	45.2	86	15.3	50	11,904
Mean for Plymouth-adjacent loggers		10.8	39.6	24.9	46.9	88	17.1	53	11,904
Plymouth, NE reported temperatures		8.8	36.7	23.7	40.3	83	13.1	42	8570
Logger 26	2	11.6	42.7	28.0	70.0	92	33.2	63	11,904
Logger 36	2	8.7	43.6	24.9	47.7	88	19.9	65	11,904
Logger 43	2	11.6	43.9	27.2	68.1	94	27.5	65	11,904
Logger 51	2	15.4	47.7	32.2	95.3	100	66.3	86	11,904
Logger 58	2	15.2	42.8	29.3	82.5	98	41.8	81	11,904
Mean Overton-adjacent loggers		12.5	44.1	28.3	72.7	94	37.7	72	11,904
Overton, NE reported temperatures		5.0	40.1	22.7	34.5	79	12.2	46	8802
Logger 46	3	11.2	43.7	26.8	66.7	90	27.4	69	11,904
Logger 53	3	11.2	40.5	27.2	67.2	94	27.1	66	11,904
Logger 57	3	22.3	36.7	29.2	92.9	99	39.3	62	11,904
Logger 60	3	9.9	37.5	24.8	46.2	82	16.2	54	11,904
Logger 52	4	16.1	38.1	23.6	29.9	57	0.5	2	11,904
Mean for Sutton-adjacent loggers		14.4	39.3	26.3	60.6	85	22.1	51	11,904
Sutton, NE reported temperatures		6.1	36.7	22.3	30.5	77	8.3	33	8822
Logger 33	6	21.0	40.0	30.1	95.3	100	47.6	88	11,904
Logger 44	6	21.7	54.4	30.6	96.5	100	52.9	92	11,904
Logger 47	6	19.8	38.8	29.7	94.0	99	44.1	82	11,904
Logger 49	6	16.2	53.4	29.3	80.3	100	4108	94	11,904
Mean for Navasota-adjacent loggers		19.7	46.7	29.9	91.5	100	46.6	89	11,904
Navasota, TX reported temperatures		18.2	41.9	29.7	85.6	100	42.4	96	8880
Logger 56	5	23.2	39.9	31.8	99.3	100	72.1	98	11,904
Logger 62	5	18.1	44.9	29.5	85.3	100	42.5	97	11,904
Logger 42	7	20.7	42.2	31.8	98.2	100	64.5	96	11,904
Logger 48	7	23.1	41.2	32.6	99.2	100	80.5	98	11,904
Logger 55	7	21.6	40.4	32.1	98.5	100	71.7	97	11,904
Logger 40	8	20.7	47.2	33.7	98.1	100	74.7	98	11,904
Logger 59	8	23.3	41.0	33.2	99.3	100	86.1	98	11,904
Logger 39	9	20.6	43.8	32.1	96.4	100	63.5	98	11,904
Mean for College Station-adjacent loggers		21.4	42.6	32.1	96.8	100	69.5	98	11,904
College Station, TX reported temperatures		17.0	41.1	28.2	99.1	100	40.1	93	3586
NE means for loggers		14.6	42.7	27.7	70.4	92	32.5	67	11,904
NE means for weather reports		6.6	37.8	22.9	35.1	80	11.2	40	8731
TX means for loggers		20.8	43.9	31.4	95.0	100	61.8	95	11,904
TX means for weather reports		17.6	41.5	29.0	92.4	100	41.3	94	6233

### Frequency and timing of excursions above the recommended storage temperatures

**Fig. 1:**
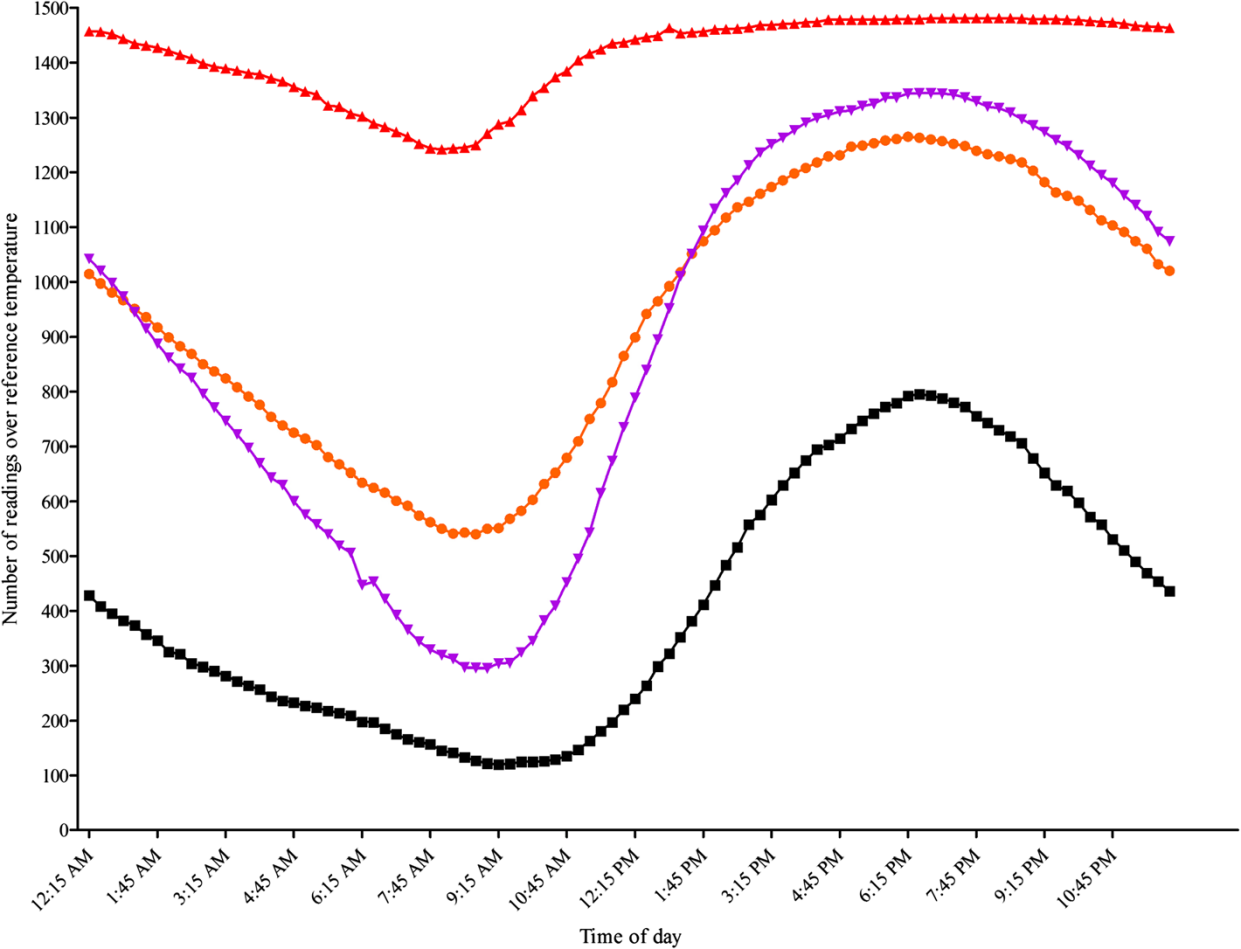
Frequency of readings exceeding the reference temperatures at each recording time point.  = Nebraska loggers with readings >25 °C;  = Nebraska loggers with readings >30 °C;  = Texas loggers with readings >25 °C;  = Texas loggers with readings >30 °C

Figure 1 demonstrates the times of day during which vehicle temperatures exceeded either of the two common recommended drug storage temperatures. The lowest number of excursions above 25 and 30 °C for Texas loggers were 1242/1488 at 8:00 AM and 295/1488 at 9:00 AM, respectively (Fig. 1). The highest number of excursions above 25 and 30 °C for Texas loggers were 1481/1488 from 6:45 to 8:30 PM and 1344/1488 from 6:30 to 6:45 PM, respectively. The lowest number of excursions above 25 and 30 °C for Nebraska loggers were 540/1488 and 120/1488, respectively, and occurred at 8:45 AM and 9:15 AM. The highest number of excursions above 25 and 30 °C for Nebraska loggers were 1265/1488 at 6:15 PM and 795/1488 at 6:30 PM.

### Ambient temperature readings

The highest daily ambient temperature for all Texas locations was 41.9 °C and for all Nebraska locations was 40.1 °C (Table 2). The mean daily temperature for Texas locations was 29.0 °C and for Nebraska was 22.9 °C with a mean daily high of 41.5 and 37.8 °C, respectively.

## Discussion

A review of the labels of 58 drugs common on large animal practice vehicles (antimicrobial, antiparasitic, anesthetic, and anti-inflammatory agents, electrolyte solutions, hormones, vitamin/mineral preparations, ophthalmic drugs, and emergency drugs) revealed that the most common maximum temperatures listed on drug inserts for storage of veterinary drugs were 25 and 30 °C. All 58 veterinary drug labels that we reviewed designated an upper limit temperature, with 47 labels citing an upper limit of 30 °C, 25 citing 25 °C and one citing 40 °C. Only three labels specifically stated that the product is to be protected from heat. There is also one product for which the tolerable temperature range changes after first puncture: the label for tildopirosin (Zuprevo, Merck Animal Health) indicates that the upper temperature limit is 30 °C, but after first puncture is reduced to 25 °C. Some pharmaceutical labels denote that temperature excursions are permitted. The labels of four surveyed products, amprolium (Corid 9.6 % Oral Solution, Merial), ceftiofur hydrochloride (Excenel RTU EZ, Zoetis), sulfamethazine (Sustain III Calf Bolus, Bimeda), and polysulfated glycosaminoglycan (Adequan i.m., Lutipold) state that temperature excursions are permitted, but no temperature or time parameters are provided on the label.

A review was also conducted of the published stability data of drugs of interest in large animal veterinary practice when stored outside the manufacturers’ storage recommendations (Table 3). Drugs with demonstrated instability when stored outside of manufacturers’ recommendations included two cephalosporin antimicrobial drugs, epinephrine, and oxytocin. No information could be found for the commonly used veterinary drugs xylazine, gonadorelin, and ceftiofur.

**Table 3:** Reported stability of drugs outside of manufacturers’ storage recommendations [11, 15–23]

Demonstrated to be stable	Equivocal data about stability	Demonstrated to be unstable
Anesthetics	Anesthetics	Cephalosporins
atropine	lidocaine	cefazolin
diazepam	naloxone	cephalothin
midazolam	Macrolides/lincosamides	Other
morphine sulfate	erythromycin	epinephrine
phenobarbital	Penicillins/aminopenicillins	heparin
Aminoglycosides	amoxicillin +/− clavulanate	oxytocin
amikacin	ampicillin	
gentamicin	penicillin G	
neomycin	Sulfonamides	
Fluoroquinolones	sulfamethoxazole	
ciprofloxacin	Tetracyclines	
difloxacin	doxycycline	
enrofloxacin	tetracycline	
marbofloxacin	Other	
norfloxacin	dopamine	
orfloxacin	furosemide	
Macrolides/lincosamides		
clindamycin		
lincomycin		
tilmicosin		
tulathromycin		
tylosin		
Penicillins		
oxacillin		
cloxacillin		
Sulfonamides		
sulfadimethoxine		
sulfamethoxazole/trimethoprim		
Tetracyclines		
chlortetracycline		
oxytetracycline		
Other		
calcium chloride		
dexamethasone		
diphenhydramine		
sodium bicarbonate		
thiamine		

Excluded drugs include extemporaneous solutions, temperature ranges significantly different than those encountered in veterinary practice, and drugs in tissues such as urine or meat

The U.S. Pharmacopeia defines controlled room temperature as 20–25 °C. Temperatures between 30 and 40 °C are considered warm, while excessive heat is defined by the U.S. Pharmacopeia to be temperatures above 40 °C [1]. For products requiring controlled room temperature storage, temperature spikes up to 40 °C are permitted if they are transient and less than 24 h in duration and if the manufacturer allows [13]. Such excursions are permitted by label presumably to allow for transient temperature spikes as may occur during shipping. Temperatures in study vehicles exceed these allowances both in magnitude and duration.

Practices and vehicles within practices were selected for inclusion in this study based on convenience rather than a random sample. Because our goal was descriptive rather than comparative, a sample size assessment was deemed unnecessary. The practice types included likely do not represent the proportion of each practice type in Nebraska or Texas. While practice type may affect the turnover of pharmaceutical inventory in storage units due to differences in the seasonality of cases, it would not be expected to influence the temperature profiles experienced in the storage units themselves.

Interestingly a majority, 18 of 24, of practice vehicles in this study were subjected to routine unshaded conditions during working hours including the time spent parked at the clinic. Conditions described as home/night were slightly better with only 10 of 24 vehicles not shaded during this time. The lack of shade for the vehicles may certainly have played a role in the temperature profiles recorded during this study.

The use of three optional features of the storage units were queried in the survey; refrigerators, day heaters, and heated water, because their use could contribute to increased temperatures within the units. Small, optional refrigerators may be purchased with the storage units and are contained within the larger storage area to store products specifically labeled for storage under refrigerated conditions. Day heaters are another optional feature of the storage units which are typically used only during periods of cold weather to heat the entire storage area to prevent exposing the stored products to unnecessarily cold or freezing temperatures. Most storage units have the capability to supply heated water for the veterinarian’s use. The storage unit’s hot water reservoir is heated by the vehicle’s cooling system if the heating system is turned on. The purpose of describing the use of these three optional features was to provide insight into relative frequency of their use and to highlight them as potential heat sources within the storage units. Analysis of the relationship of these features and the temperatures within the storage units was outside the scope of this study.

The study time period, May 15 to September 15, was selected to coincide with the summer months to provide the highest potential temperature exposures and in the experience of the authors, a time of year in many large animal practices when practice vehicle pharmaceutical inventory experiences a slow turnover due to decreased case loads. The results of the study, therefore, may represent a worst case scenario for pharmaceutical storage.

As expected the highest temperatures recorded in the storage units were recorded at locations in Texas. However, mean temperatures recorded by Nebraska temperature loggers were over the 25 °C labelled upper limit storage temperature of many commonly used pharmaceuticals. Of even more concern is that the mean temperatures recorded by Texas temperature loggers were over the 30 °C labelled upper limit storage temperature of certain pharmaceuticals. Additionally, a large number of days with at least one recorded temperature exceeding the reference temperatures and a large number of individual recordings exceeding the reference temperatures were recorded in both states. These findings indicate pharmaceuticals maintained in practice vehicle’s storage units were exposed to temperatures above their labelled storage range a significant portion of the time during the summer months in both Nebraska and Texas. These findings are consistent with a similar study in Austria which evaluated drug-compartment temperatures in car, van, and utility veterinary practice vehicles [14].

The frequency of excursions above 25 and 30 °C for Texas and Nebraska loggers as shown in Fig. 1 followed a consistent pattern with the lowest frequency occurring in the mid-morning hours and highest frequency of excursions occurring in the early evening hours. However, it is important to note that multiple excursions over both reference temperatures occurred at all of the 96 daily time points. This indicates the need for implementation of practices which will reduce heat accumulation in portable veterinary units throughout the day, but particularly during the late afternoon and early evening hours.

Analysis of the relationship between ambient temperatures and temperatures within the storage boxes was outside the objective of this study. Local ambient temperatures were provided to serve as a reference to the conditions encountered by the storage units. However, it is interesting to note the temperatures recorded in the storage units consistently exceeded the environment temperatures suggesting a greenhouse effect in the storage unit which may have been exacerbated by a lack of shade.

## Conclusions

Temperatures in drug storage units in participating Nebraska and Texas veterinary practice vehicles routinely exceeded labelled drug storage temperatures. Vehicles were routinely left unprotected from direct sunlight and utilized one or more optional features which may have contributed to these findings. More research is needed to determine whether these excursions alter efficacy of stored drugs or lead to degradation products which may pose a health risk to the patient or eventually the consumer. However, until more data are available, veterinarians should consider whether maintaining drug inventories above manufacturers’ labeled temperatures may leave them at risk of liability for potential consequences of using drugs stored under these conditions. Furthermore, veterinarians should consider providing shade to their practice vehicles and employing judicious use of optional features such as intra-unit refrigerators, day heaters, and heated water while monitoring the temperatures within their storage units to reduce the occurrence of excursions over the labelled storage temperatures.

## Abbreviations

U.S.: United States
USP: United States Pharmacopeia

## Footnotes

1 www.wunderground.com
